# How to use large language models in ophthalmology: from prompt engineering to protecting confidentiality

**DOI:** 10.1038/s41433-023-02772-w

**Published:** 2023-10-05

**Authors:** Oliver Kleinig, Christina Gao, Joshua G. Kovoor, Aashray K. Gupta, Stephen Bacchi, Weng Onn Chan

**Affiliations:** 1 Royal Adelaide Hospital, Adelaide, SA Australia; 2 University of Adelaide, Adelaide, SA Australia; 3 Gold Coast University Hospital, Gold Coast, QLD Australia; 4 Flinders University, Adelaide, SA Australia

**Keywords:** Education, Medical research

## Introduction

Large language models (LLM) are complex predictive text algorithms that generate sentences and paragraphs from a prior sequence of words [1]. With appropriate training, they can act conversationally by generating text following a user-provided instruction or question. Commonly known modern LLMs include OpenAI’s GPT-3 and GPT-4, Google’s PaLM-2, and Meta’s Llama-2. These models vary in design and performance. Information on accessing these models is included in the supplementary information.

Each implementation of the same LLM (e.g. GPT-4) may behave differently due to differences in ‘fine-tuning’ training, plugin access [2], and initial instructions [3]. Early evidence suggests, when answering ophthalmology examination questions, GPT-4 via the ChatGPT or New Bing chatbot is the most performant [2, 4–6]. Understanding how to effectively employ LLMs may have significant utility for clinical education and, in future, clinical practice.

## What can LLMs do?

LLMs may be effective in language tasks like writing, editing, and summarizing. For querying, LLMs excel at questions that are not easily web-searchable, or fact-finding without searchable keywords. LLMs can also be queried to write code in R and other languages. LLMs perform mathematical operations inaccurately [7]. Plugins like embedded calculators (Supplementary Information) can address specific LLM limitations.

## Engaging LLMs

LLMs are engaged through text input. Slight variations in input text can cause significant variations in output [8]. Resultingly, high-quality prompting, or ‘prompt engineering’, is critical in optimizing LLM outputs.

### Asking the right question

It is widely accepted that providing specific instructions to LLMs yields more useful outputs [9]. For example, requesting a summary of a condition’s epidemiology, pathophysiology, diagnosis, and treatment is generally superior to asking for general information (Box 1). Additionally, using specifiers such as “Provide a brief/detailed summary of”, “Explain at a high-school/college-grade/consultant level”, and “Present your findings in a table/as dot points” sets specific parameters to allow LLMs to generate more useful outputs.

Box 1 The different outputs from ChatGPT (July 20 GPT-4 version) when asking “Briefly describe conjunctivitis” and “Briefly describe the epidemiology, pathophysiology, diagnosis, and treatment of conjunctivitis”

**Table:** 

Query	Briefly describe conjunctivitis.	Briefly describe the epidemiology, pathophysiology, diagnosis, and treatment of conjunctivitis.
Response	Conjunctivitis, often referred to as “pink eye”, is an inflammation or infection of the conjunctiva, the transparent membrane that lines the inner part of the eyelids and covers the white part of the eyes. This condition can be caused by several factors: 1. Bacterial or viral infections: These are the most common causes, often resulting from a cold or other respiratory infection. These forms of conjunctivitis are contagious and can spread easily. 2. Allergies: Allergic conjunctivitis happens when the body reacts to an allergen, such as pollen, dust mites, or animal dander, and is not contagious. 3. Irritants: Exposure to irritants like chlorine in swimming pools, smog, smoke, or certain cosmetics can also lead to conjunctivitis. Common symptoms include redness of the eyes, increased tear production, a gritty feeling in the eyes, itchiness, and a discharge that can form a crust during sleep. Treatment varies based on the cause. Bacterial conjunctivitis can be treated with antibiotics, while viral conjunctivitis often resolves on its own. Allergic conjunctivitis might be treated with antihistamines, and conjunctivitis from irritants usually clears up by avoiding the triggering factor.	**Epidemiology:** Conjunctivitis, also known as pink eye, is a common condition worldwide. Its incidence varies based on the cause, whether it’s viral, bacterial, allergic, or due to other causes like chemical exposure or certain diseases. Viral conjunctivitis is often associated with respiratory infections and can spread easily in close-knit communities, such as schools and nursing homes. Bacterial conjunctivitis is often seen in children and may occur with an ear infection. Allergic conjunctivitis is linked to seasonal allergies or exposure to specific allergens. **Pathophysiology:** The conjunctiva is a clear tissue covering the white part of the eye and the inside of the eyelids. Conjunctivitis is the inflammation of this tissue. Depending on the cause, the pathophysiology differs. In bacterial and viral conjunctivitis, the pathogens invade and irritate the conjunctiva, causing inflammation. In allergic conjunctivitis, exposure to allergens triggers an immune response, leading to inflammation. Chemical or irritant conjunctivitis occurs when the conjunctiva is irritated by a foreign substance. **Diagnosis:** Conjunctivitis is often diagnosed based on a clinical examination and patient history. Symptoms can include redness in the white of the eye, increased tear production, a feeling of grittiness in the eye, itching, burning, blurred vision, and an increased sensitivity to light. A patient’s recent history of a cold, exposure to a person with pink eye, or a history of allergies can also provide clues. In some cases, a sample of the discharge from the eye may be taken for laboratory analysis to confirm the diagnosis and to differentiate the cause. **Treatment:** Treatment for conjunctivitis depends on the cause. Viral conjunctivitis typically resolves on its own without treatment, although cool compresses and artificial tears can help relieve symptoms. Bacterial conjunctivitis is treated with antibiotic eye drops or ointments. Allergic conjunctivitis is managed by avoiding the allergen, if possible, and with antihistamines or anti-inflammatory medications. For irritant conjunctivitis, the eye is often rinsed to remove the irritant, and then cool compresses and artificial tears can be used to soothe the eye. In all cases, maintaining good eye hygiene is crucial to prevent the spread of infection. Always consult a healthcare professional if you suspect you have conjunctivitis, as some forms can be serious and require medical intervention.

### Preparing the LLM

‘Priming’ LLMs for expected inputs and responses is an emerging practice [2]. For example: “I am going to provide X; I want you to output Y”. This strategy increases the chance of a useful output. Example outputs can also be offered to LLMs to style-mimic. Online libraries of example ‘guiding prompts’ are emerging (for example https://www.learnprompt.org/act-as-chat-gpt-prompts/).

Notably, certain instructions could worsen output quality. ChatGPT’s outputs are most accurate after explaining its reasoning [10]. Therefore, prompts that explicitly request LLMs omit reasoning could result in lower-quality outputs.

Additionally, during the same session, LLMs will consider previous inputs and outputs in response [7]. Given their text-completion ‘urge’, LLMs will tend to mimic the format of prior outputs. This property may lower user workload as guiding prompts need only be typed once. Beginning a new session is prudent for unrelated conversations to reduce memory retention bias from prior outputs [5, 11].

It is also an emerging practice to tell LLMs, “If you are not sure about the answer, say you don’t know”. These uncertainty prompts may reduce, but not prevent, inaccurate outputs in certain tasks [12].

### Completing tasks

As LLMs are effective information summarizers, they may streamline the non-creative parts of ophthalmological writing, such as abstract creation [13, 14] or medical letter generation [15]. However, ChatGPT may omit or hallucinate information [14, 15], so human oversight is required [15]. Additionally, the creative components of medical writing (for example, drawing conclusions from data) are most accurately performed by a credible human [13].

As these models are pre-trained to complete text, LLMs occasionally fail to summarize or edit incomplete documents. This is due to the ‘urge’ to complete the inputted text. In these situations, it is best to provide LLMs with clearly fragmented or completed text.

### Powerful prompts

Powerful prompts guide LLMs in higher-order tasks, such as requesting more information for a conclusion. An LLM employed to diagnose ophthalmological conditions could be asked: “Patient X is [known information]. Keep asking me questions about patient X until you have sufficient information to arrive at a diagnosis.” This method can minimize uncertainty and reduce the generality of responses provided by an LLM.

Another powerful prompt pattern is asking LLMs for feedback. For example, it is possible to ask LLMs for feedback on a patient handout and then request the LLM implement such feedback, as shown in Box 2. This process can be iterated multiple times.

Lastly, when unsure of how to prompt an LLM, it is possible to ask LLMs like ChatGPT to ask clarifying questions about a desired task until it has sufficient information to write its own prompt. The supplementary information includes an example prompt-generating prompt [16].

Box 2 Using ChatGPT (GPT-4, August 3 Version) to create an iterate on a patient handout for cataracts surgery

**Table:** 

Prompt	Create me a patient handout for cataracts surgery.
Response	**Cataract Surgery: Patient Handout** What is a Cataract? A cataract is a clouding of the eye’s natural lens, which lies behind the iris and the pupil. Cataracts are the most common cause of vision loss in people over the age of 40 and are the principal cause of blindness worldwide. What is Cataract Surgery? Cataract surgery is a procedure to remove the lens of your eye and, in most cases, replace it with an artificial lens. When Is Surgery Recommended? - Difficulty in reading, driving, or performing daily tasks - Vision impairment that cannot be corrected with glasses - Decreased night vision **Preparing for Surgery** Pre-operative Tests - Eye measurement tests - General health evaluation Medication - Inform your doctor about all medications you are taking - You may be advised to stop certain medications Fasting - Typically, you will be asked to fast for 6–12 h before surgery **The Procedure** Duration - Usually 15–20 min per eye Steps 1. *Local Anesthesia*: Eye drops to numb the area 2. *Small Incision*: Cut near the cornea 3. *Lens Removal*: Broken up and vacuumed out 4. *Artificial Lens Insertion*: A foldable lens is inserted **After Surgery** Immediate Care - You’ll be observed for complications for a short period - You may wear a protective eye shield or patch Medication - Antibiotic eye drops - Anti-inflammatory eye drops Activity - Avoid strenuous activities for a few weeks - No swimming for up to two weeks - Avoid rubbing your eyes **Risks and Complications** - Infection - Bleeding - Retinal detachment - Secondary cataract - Pressure increase inside the eye **Follow-Up** - One day after surgery - One week after surgery - One month after surgery **FAQs** Q: Is the procedure painful? A: Most patients report minimal discomfort, often describing a sensation of pressure. Q: When will my vision improve? A: Many people report clear vision within several hours, but each person heals differently. Q: Can cataracts come back? A: No, but a condition called “secondary cataract” might occur, which can be easily treated. **Important Contacts** *Surgeon*: Dr. [Name] *Contact*: [Contact Information] Please consult your healthcare provider for personalized medical advice. **Disclaimer**: This handout is for informational purposes only and should not be considered as medical advice.* — We hope this handout serves as a helpful guide for you as you prepare for cataract surgery. Feel free to ask your healthcare provider any additional questions you may have.
Prompt	Give me specific and targeted feedback on this handout.
Response	Certainly! The handout is well-structured and provides a comprehensive overview of what a patient needs to know about cataract surgery. However, there are some areas where it could be improved for clarity, depth, and engagement: **Strengths:** 1. *Structured Layout*: The layout is well-organized, making it easy to follow. 2. *Use of Subheadings*: Subheadings help to break down the text into manageable sections. 3. *FAQs*: This is a valuable addition as it anticipates patient concerns. **Areas for Improvement:** 1. *Terminology*: While the handout uses relatively simple language, medical terms like “Retinal detachment” or “Secondary cataract” may need explanations for the layperson. 2. *Visual Aids*: The inclusion of diagrams or images could make the information easier to digest, particularly for visual learners. For instance, a diagram of the eye could be useful for explaining what a cataract is. 3. *Personalization*: Including a section where patients could jot down their own questions for the doctor might make the handout more interactive and personalized. 4. *Preparation Details*: While you mention pre-operative tests, it might be useful to list common tests like a ‘slit-lamp examination’ or ‘visual acuity test’ so patients know what to expect. 5. *Post-Op Symptoms*: A brief section on what normal and abnormal post-op symptoms look like could prepare patients for the recovery phase. 6. *Emergency Contacts*: Adding a section for emergency contact details could be beneficial for immediate post-op care. 7. *Disclaimers and Citations*: You include a general disclaimer, but it might be beneficial to cite sources or guidelines that the handout is based on. 8. *Contact Information*: Expand this section to include not just the surgeon’s contact but also a helpline or nurse’s station for post-op concerns. 9. *Risks and Complications*: This section could be more detailed. It would be beneficial to include how often these complications occur and what steps are taken to mitigate them. 10. *Checklist*: A pre-operative and post-operative checklist could be a good addition. Patients could use it to track their preparations and recovery. 11. *Downloadable Resources*: If this handout is also made available online, links to further reading or video explanations could be included. By addressing these points, the handout can offer more depth while still remaining accessible to patients. Overall, it serves as a solid foundation and could be even more informative and engaging with these enhancements.
Prompt	Implement this feedback on the above patient handout
Response	**Cataract Surgery: Patient Handout** What is a Cataract? A cataract is a clouding of the eye’s natural lens, which lies behind the iris and the pupil. Cataracts are the most common cause of vision loss in people over the age of 40 and are the principal cause of blindness worldwide. [Diagram of the Eye Showing Cataract Location] What is Cataract Surgery? Cataract surgery is a procedure to remove the lens of your eye and, in most cases, replace it with an artificial lens. When Is Surgery Recommended? - Difficulty in reading, driving, or performing daily tasks - Vision impairment that cannot be corrected with glasses - Decreased night vision **Preparing for Surgery** Pre-operative Tests - Slit-lamp Examination - Visual Acuity Test - Eye measurement tests Medication - Inform your doctor about all medications you are taking - You may be advised to stop certain medications Fasting - Typically, you will be asked to fast for 6–12 h before surgery **The Procedure** Duration - Usually 15–20 min per eye Steps 1. *Local Anesthesia*: Eye drops to numb the area 2. *Small Incision*: Cut near the cornea 3. *Lens Removal*: Broken up and vacuumed out 4. *Artificial Lens Insertion*: A foldable lens is inserted **After Surgery** Immediate Care - You’ll be observed for complications for a short period - You may wear a protective eye shield or patch Medication - Antibiotic eye drops - Anti-inflammatory eye drops Activity - Avoid strenuous activities for a few weeks - No swimming for up to two weeks - Avoid rubbing your eyes Symptoms to Watch - Normal: Mild discomfort, cloudy vision - Abnormal: Severe pain, vision loss **Risks and Complications** - *Infection*: Rare but serious. Immediate medical attention needed. - *Bleeding*: Rare and usually minor. - *Retinal Detachment*: Separation of retina from the back of the eye; requires immediate attention. - *Secondary Cataract*: Cloudiness that can occur months or years later; can be easily treated. - *Pressure increase inside the eye*: May require medication. **Follow-Up** - One day after surgery - One week after surgery - One month after surgery **FAQs** Q: Is the procedure painful? A: Most patients report minimal discomfort, often describing a sensation of pressure. Q: When will my vision improve? A: Many people report clear vision within several hours, but each person heals differently. Q: Can cataracts come back? A: No, but a condition called “secondary cataract” might occur, which can be easily treated. **Important Contacts** *Surgeon*: Dr. [Name] *Contact*: [Contact Information] *Nurse’s Station*: [Contact Information] *Emergency Contact*: [Contact Information] **Your Notes** - [Your questions for the doctor] - [Your symptoms post-surgery] - [Your medication schedule] **Checklists** Pre-Op Checklist - [ ] Completed all tests - [ ] Confirmed medications - [ ] Started fasting Post-Op Checklist - [ ] Follow-up appointments scheduled - [ ] Medication taken as advised - [ ] Activity restrictions followed *Disclaimer: This handout is for informational purposes only and should not be considered as medical advice. It is based on current medical guidelines as of [Year].* — We hope this revised handout serves as a helpful guide for you as you prepare for cataract surgery. Feel free to ask your healthcare provider any additional questions you may have. For further reading, you can visit [Resource Link]. — **Sources and Guidelines**: [Source 1], [Source 2], [Source 3]

*This process can be iterated multiple times to further refine the handout.

**Note the outputs have been altered slightly for formatting purposes, no changes to content were made.

The LLM is first asked to create the patient handout. Then the LLM is prompted to give targeted feedback on the above handout. Lastly, the LLM is tasked with implementing such feedback.

### Online or local

Critically, data provided to online LLMs such as ChatGPT and Bard may be periodically reviewed by humans [17] or be used to train models [18]. Due to this insecurity, confidential data, even deidentified, should *never* be shared with an online LLM [19]. It is possible to run some LLMs on-device, for example GPT4ALL (https://gpt4all.io) and Llama-2 (https://ai.meta.com/llama/). However, this may result in an unfavorable trade-off between model capability and speed on consumer-grade systems. Consequently, enterprise-hosted and institutionally and ethics committee approved LLMs may be the most appropriate LLM implementation for analysis of confidential information.

## What limits LLMs?

While powerful tools, LLMs have well-documented limitations [20]. Most significantly being inaccuracies [20], potential lack of knowledge of specialist fields and, for GPT-3 and GPT-4, knowledge of events and advancements following September 2021 [7]. Furthermore, LLM’s performance may vary; ChatGPT’s capability (GPT-3 and GPT-4) declined in early 2023 [21], potentially due to increased safety training [10]. In the future, less safe models explicitly trained for professionals, such as Med-PaLM-2, may allow ophthalmologists to access more powerful LLMs.

## Conclusion

The use of LLMs in ophthalmology is emerging and largely untested. Understanding the function of an LLM as a powerful but fallible predictive text algorithm may guide optimal use of this technology. When using LLMs, choosing deliberately powerful prompts such as the forms suggested above will result in the highest quality use of LLMs in ophthalmology.

### Supplementary information

Supplemental Material
