# Supplementary material for: How to use large language models in ophthalmology: from prompt engineering to protecting confidentiality
Source: Eye (Lond). 2023 Oct 5;38(4):649–53. doi: 10.1038/s41433-023-02772-w (PMC10920651; doi:10.1038/s41433-023-02772-w)
Supplement: Supplementary file 1 — Supplemental Material [file 41433_2023_2772_MOESM1_ESM.docx]

**Supplementary Information**

Accessing LLMs

OpenAI’s models are publicly available as ChatGPT (<https://chat.openai.com>), New Bing, and through other services. Google’s PaLM-2 is used to power the chatbot Bard (<https://bard.google.com>).

ChatGPT can be accessed through creating an account on the ChatGPT website (<https://chat.openai.com>). Accessing the GPT-4 LLM currently requires a USD 22 per month subscription. The GPT-3 model is available for free. New Bing can be accessed by downloading the Edge search engine and clicking the New Bing icon in the top right corner. Bard is available at <https://bard.google.com>.

Using extensions

Some LLMs contain extensions on their abilities, for example web search access, the ability to use a calculator, and even the ability to access catalogues of hotels and flights. It is prudent to choose an LLM that aligns with the required capabilities of a given task. For example, if up-to-date information is required, selecting an LLM with web search access, such as New Bing or Google Bard, may yield the highest quality answers.

A prompt that can be given to LLMs like ChatGPT to get them to create their own prompts.

“I want you to become my Prompt Creator. Your goal is to help me craft the best possible prompt for my needs. The prompt will be used by you, ChatGPT. You will follow the following process: 1. Your first response will be to ask me what the prompt should be about. I will provide my answer, but we will need to improve it through continual iterations by going through the next steps. 2. Based on my input, you will generate 3 sections. a) Revised prompt (provide your rewritten prompt. it should be clear, concise, and easily understood by you), b) Suggestions (provide suggestions on what details to include in the prompt to improve it), and c) Questions (ask any relevant questions pertaining to what additional information is needed from me to improve the prompt). 3. We will continue this iterative process with me providing additional information to you and you updating the prompt in the Revised prompt section until it’s complete.”
